# NAG-1/GDF15 Transgenic Mouse Has Less White Adipose Tissue and a Reduced Inflammatory Response

**DOI:** 10.1155/2013/641851

**Published:** 2013-05-13

**Authors:** J. M. Kim, J. P. Kosak, J. K. Kim, G. Kissling, D. R. Germolec, D. C. Zeldin, J. A. Bradbury, S. J. Baek, T. E. Eling

**Affiliations:** ^1^Laboratory of Molecular Carcinogenesis, National Institutes of Environmental Health Sciences, NIH, Research Triangle Park, NC 27709, USA; ^2^Division of Structural and Functional Genomics, Korea National Institute of Health, Osong 363-951, Republic of Korea; ^3^Biostatistics Branch, National Institutes of Environmental Health Sciences, NIH, Research Triangle Park, NC 27709, USA; ^4^National Toxicology Program, National Institutes of Environmental Health Sciences, NIH, Research Triangle Park, NC 27709, USA; ^5^Laboratory of Respiratory Biology, National Institutes of Environmental Health Sciences, NIH, Research Triangle Park, NC 27709, USA; ^6^Department of Biomedical and Diagnostic Sciences, College of Veterinary Medicine, University of Tennessee, Knoxville, TN 37996, USA; ^7^Eicosanoid Biochemistry Section, Laboratory of Molecular Carcinogenesis, 111 T.W. Alexander Drive, Research Triangle Park, NC 27709, USA

## Abstract

NAG-1/GDF15 is a TGF-**β** superfamily member with poorly characterized biological activity proposed to inhibit inflammatory cytokine production. Transgenic mice expressing human NAG-1/GDF15 (*NAG*-1^*Tg*/*Lox*^) are leaner with lower body weight and are resistant to chemically or genetically induced intestinal tumors. Because of the link between obesity, inflammation, and cancer, we examined whether these mice exhibit a reduced response to inflammatory stimuli. The *NAG*-1^*Tg*/*Lox*^ mice had a reduced inflammatory response to LPS based on the serum levels of cytokines KC, IL-6, MCP-1, and TNF**α**. In contrast to literature reports and our *in vivo* results, NAG-1 did not inhibit LPS-induced cytokine expression *in vitro* in RAW264.7 cells, mouse peritoneal macrophages, or mouse liver Kupffer cells, suggesting that NAG-1/GDF15 does not directly inhibit LPS-induced inflammatory cytokine production. However, *NAG*-1^*Tg*/*Lox*^ mice have less white adipose tissue, the major source of inflammatory adipokines including leptin. Basal and LPS-treated serum leptin and mRNA levels in the adipose tissue of *NAG*-1^*Tg*/*Lox*^ mice were lower than those in WT mice. We propose that the reduced white adipose tissue and reduced leptin expression may be responsible, in part, for the reduced inflammatory response to LPS and the decrease in intestinal tumors observed in *NAG*-1^*Tg*/*Lox*^ mice.

## 1. Introduction

Nonsteroidal anti-inflammatory drug-activated gene (NAG-1/GDF15) is a divergent member of the TGF-*β* superfamily and is also known as macrophage inhibitory cytokine (MIC-1) [1, 2]. NAG-1/GDF15 expression is induced by a number of anti-inflammatory drugs and chemicals known to have antitumorigenic activities, including cyclooxygenase inhibitors, dietary agents, PPAR agonist, and anticancer drugs [3]. The overexpression of NAG-1 in cancer cells stimulates apoptosis and inhibits tumor growth in mouse xenograft models [3]. A transgenic mouse was developed in this laboratory which ubiquitously expresses human NAG-1/GDF15 (*NAG*-1^*Tg*/*Lox*^) under the control of the chicken *β*-actin promoter (CAG) [4]. In addition to expression in most tissues including skin, kidney, colon, brain, and lung, it is found at high levels in the serum (~50 ng/mL), as NAG-1 is a secreted protein. The mouse has no apparent phenotype except for being leaner with DEXA densitometry (PIXImus) analysis indicating less adipose tissue. Previously we reported that the transgenic mice are resistant to chemically and genetically induced intestinal tumors [4]. Treatment of the transgenic mice with the intestinal carcinogen azoxymethane (AOM) induced fewer intestinal tumors as compared to the wild-type (WT) mice. Crossing the NAG-1 transgenic mouse with the *APC* ^min⁡^ mouse that spontaneously develops polyps produced an *APC* ^min⁡^ mouse expressing NAG-1. This mouse developed fewer polyps than the WT *APC* ^min⁡^ mouse. These data support the notion that NAG-1 acts to suppress intestinal tumor formation during the early stages of tumorigenesis. Because overexpression of NAG-1 induces apoptosis in cultured cancer cells [5], we expected increased apoptosis to be one of the mechanisms for the inhibition of intestinal tumor growth in our mouse model. However, we did not observe spontaneous apoptosis in the *NAG*-1^*Tg*/*Lox*^ mouse, suggesting other mechanisms are responsible for the tumor suppression. 

Inflammation can be an important determinant in the development of cancer and is a driving force for the development of colorectal cancer [6, 7]. Many reports in the literature confirm the importance of inflammation in AOM-induced intestinal tumor formation. For example, genetic deletion of critical NFkB components reduces tumor formation [8] in mice treated with AOM. Inflammation also plays a critical role in the *APC* ^min⁡^ mouse model [9, 10]. Bootcov et al. [11] previously reported that NAG-1/GDF15 inhibits LPS-induced cytokine formation in macrophages, the experimental basis for naming the protein MIC-1 (macrophage inhibitory cytokine). Thus, we suspected one potential mechanism for the resistance of the *NAG*-1^*Tg*/*Lox*^ to intestinal tumor formation was a lower inflammatory response. 

In humans, the accumulation of white adipose tissue in obesity provides a source of chronic inflammation, which likely explains the fact that obese people are at increased risk for developing colorectal cancer [12]. Various mouse models have highlighted the pivotal role of white adipose-derived leptin as an inflammatory cytokine that plays a critical role in the development of intestinal cancer. Leptin is a secreted adipokine, whose levels are proportional to one's amount of white adipose tissue [13]. Obesity increases serum leptin levels, which acts as a growth and survival factor for colon epithelial cells and increases tumor growth in the AOM model [14]. Mice lacking the leptin gene (*ob* ^−^/*ob* ^−^) [14] have very high levels of white adipose tissue but are devoid of leptin expression and have a lower tumor burden after treatment with AOM. Furthermore, obese leptin receptor deficient (*db* ^−^/*db* ^−^) mice have decreased intestinal tumor formation after AOM treatment. Obese KK-A^y^ mice are highly susceptible to AOM-induced colorectal cancer [15], exhibit high serum leptin concentrations, and increased expression of inflammatory cytokines in their serum and visceral fat. These findings support the importance of leptin as an inflammatory link between white adipose tissue and intestinal tumorigenesis. 

In this report, we test whether the *NAG*-1^*Tg*/*Lox*^ mouse exhibits a reduced response to inflammatory stimuli, to help explain its protection against tumor formation in the AOM and *APC* ^min⁡^ models of intestinal tumorigenesis. The *NAG*-1^*Tg*/*Lox*^ mice show a reduced systemic inflammatory response to lipopolysaccharide (LPS), exhibited by lower levels of inflammatory cytokines in their serum. Interestingly, this reduction was not due to direct inhibition of cytokine production by NAG-1 in macrophages. *NAG*-1^*Tg*/*Lox*^ mice are leaner [4] and have significantly lower amounts of white adipose tissue (WAT) and thus lower levels of leptin. We propose that the smaller WAT and hence lower leptin expression may be responsible for the lower inflammatory response and lower intestinal polyp formation observed in the *NAG*-1^*Tg*/*Lox*^ mouse. This study illustrates how white adipose tissue can alter inflammation. 

## 2. Methods and Procedures

### 2.1. Animals

As described previously, we produced two independent transgenic mouse lines (1377 and 1398) that ubiquitously express human NAG-1 protein, *NAG*-1^*Tg*/*Lox*^, under the control of CAG, using traditional pronuclear microinjection on the C57/BL6 background [4]. Seven-to twelve-week-old male and female mice from lines 1377 and 1398 *NAG*-1^*Tg*/*Lox*^ mice and NAG-1 wild-type (WT) littermates were used in these experiments. The mice were housed at NIEHS or ILS under guidelines for the ethical treatment of animals. All procedures conducted in this study were approved by the appropriate institutional animal care and use committees. 

### 2.2. LPS Treatment and Cytokine Analysis

*NA* *G*-1^*Tg*/*Lox*^ mice and nontransgenic WT littermates of both 1377 and 1398 lines were treated with LPS (25 mg/kg, Sigma) via intraperitoneal (i.p.) injection and then sacrificed by CO_2_ inhalation and the blood was collected at the times indicated in the figure legends. The serum was used for cytokine analysis using the Multiplex cytokine detection assay system according to the manufacturer's instructions (Bio-Plex mouse cytokine assay, Bio-Rad). 

### 2.3. Real-Time Polymerase Chain Reaction and Primers

Total RNA was isolated using the RNeasy Mini Kit (Qiagen) according to the manufacturer's instructions. Following DNAse I treatment (Invitrogen), reverse transcription was carried out with 1 *μ*g total RNA using the SuperScript II reverse transcription system (Invitrogen) according to the manufacturer's instructions. Real-time PCR was performed in duplicate with individual time-matched vehicle-treated controls using SYBR Green PCR Master Mix (BioRad), a BioRad iCycler Thermal Cycler and the following primers:

 hNAG-1, F: 5′-ctccagattccgagagttgc-3′, R: 5′-agagatacgcaggtgcaggt-3′
 mGDF15, F: 5′-ttctgtggggacggtcag-3′, R: 5′-cgggtgaccaggctaattc-3′
 mCOX-2, F: 5′-cccccacagtcaaagacact-3′, R: 5′-gagtccatgttccaggagga-3′
 mMcp-1, F: 5′-aggtccctgtcatgcttctg-3′, R: 5′-tctggacccattccttcttg-3′
 mKC, F: 5′-gcacccaaaccgaagtcata-3′, R: 5′-tggggacaccttttagcatc-3′
 mIL-6, F: 5′-ccggagaggagacttcacag-3′, R: 5′-ggaaattggggtaggaagga-3′
 mTNF-*α*, F: 5′-cgtcagccgatttgctatct-3′, R: 5′-cggactccgcaaagtctaag-3′
 mLeptin, F: 5′-tgacaccaaaaccctcatca-3′, R: 5′-tgaagcccaggaatgaagtc-3′
 mLeptin Receptor, F: 5′-gggaatgagcaaggtcaaaa-3′, R: 5′-tcaagtcccctttcatccag-3′
 mIL-1*β*, F: 5′-caggcaggcagtatcactca-3′, R: 5′-tgtcctcatcctggaaggtc-3′
 mGAPDH, F: 5′-caggagcgagaccccactaacat-3′, R: 5′-gtcagatccacgacggacacatt-3′
 m*β*-actin, F: 5′-tgacaggatgcagaaggaga-3′, R: 5′-cgctcaggaggagcaatg- 3′.

The following cycle parameters were used: 95°C for 10 minutes; 40 cycles of 95°C for 15 seconds, 60°C for 30 seconds; 95°C for 1 minute; 60°C for 1 minute; 81 cycles of 55°C for 10 seconds. BioRad iQ5 Version 2.0 software was used to calculate Ct values for all genes. Corrected Ct values were calculated by subtracting the average control gene (actin or GAPDH) Ct value from the average Ct value for a given gene. Fold differences were calculated using the corrected Ct values. 

### 2.4. RAW Cell Culture and Transfections

RAW 264.7 (ATCC) cells were plated in 60 mm culture dishes and grown overnight in DMEM supplemented with 10% fetal bovine serum. The next day, the cells were transfected with either empty vector or an NAG-1 expression vector [16] using Lipofectamine 2000 according to the manufacturer's protocol (Invitrogen) for 24 hours. Cells were treated with LPS (1 *µ*g/mL) or vehicle for 6 hours. The transfections were performed for three independent times. RNA isolation and RT-PCR were performed as described previously. 

### 2.5. Isolation and Culture of Intraperitoneal Macrophages and Kupffer Cells

To obtain intraperitoneal macrophages, mice were injected i.p. with 3% thioglycollate solution to enhance macrophage recruitment and 3 days later peritoneal cells were isolated. Kupffer cell isolation was performed following *Current Protocols in Toxicology *[17]. Briefly, mouse livers were perfused by inserting and securing a catheter through the vena cava, severing the portal vein and perfused with prewarmed CMF-HBSS until clear, then pre-warmed 0.03% collagenase type IV in HBSS at a rate of 5 mL/min for 5 minutes. Perfused livers were kept on ice in a small volume of CMF-HBSS in sterile conical polypropylene tubes until all tissue collection was completed. To obtain adequate cell numbers, the livers of two mice per genotype were perfused and pooled prior to Kupffer cell isolation. After Percoll gradient centrifugation, pellets were resuspended in 1 ml HBSS and counted using an automated cell counter (Nexcelom Bioscience). Consistently, from both WT and *NAG*-1^*Tg*/*Lox*^ mice, 1-2 million Kupffer cells were isolated per mouse liver. Equal numbers of either WT or *NAG*-1^*Tg*/*Lox*^ Kupffer cells were plated in triplicate onto 6-well dishes containing RPMI 1640 with 10% FBS, cultured for 24 hours in a humidified incubator at 37°C containing 5% CO_2_, then treated with 10 ng/mL LPS or vehicle for 6 hours prior to harvest. Cell culture media were collected, spun briefly to remove cell debris, aliquoted, and frozen at –80°C until ELISA analysis was performed. In experiments using the recombinant protein, WT Kupffer cells were treated with either 50 ng/mL recombinant human GDF-15 (SBH Sciences, MA, USA) or vehicle for 2 hours prior to LPS treatment. 

### 2.6. Recombinant NAG-1 Protein

Purified recombinant human NAG-1 was purchased from R&D Systems (Minneapolis, MN, USA) and from SBH Sciences (Natick, MA, USA). The R&D Systems protein contains an N-terminal 6-His tag, whereas the SBH Sciences protein does not. The lyophilized protein was reconstituted with 1% BSA in PBS and stored at 4°C. 

### 2.7. Statistical Analysis

Student's *t* test and standard deviation (SD) were calculated using Microsoft Excel (Microsoft, Redmond, WA, USA). Standard error was calculated with Stat View (Abacus Concept, Berkeley, CA, USA). Statistical analysis of cytokine levels in sera was done by analysis of variance (ANOVA). 

## 3. Results 

### 3.1. NAG-1 Transgenic Mice Have a Decreased Inflammatory Response to LPS

**Figure 1:**
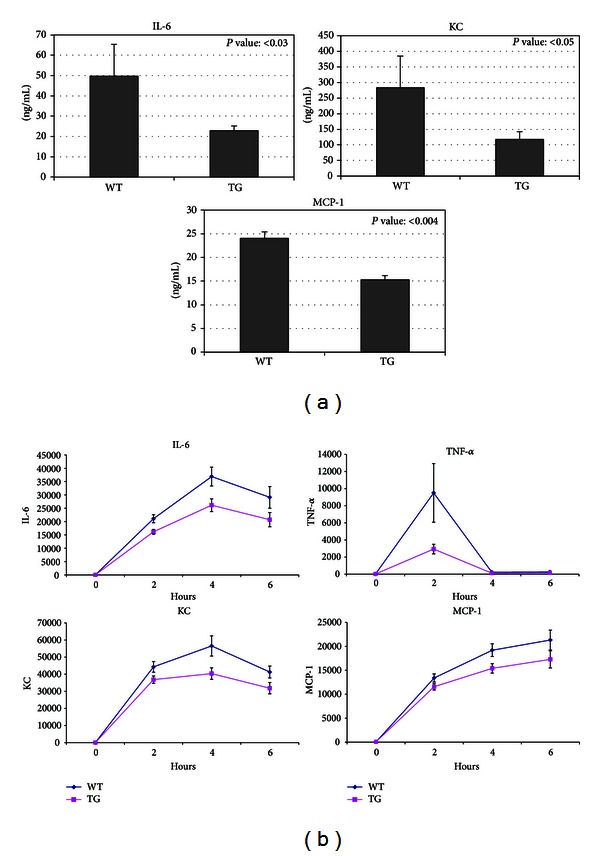
NAG-1 transgenic mice have a decreased inflammatory response to LPS. Seven- to twelve-week-old *NAG*-1^*Tg*/*Lox*^ mice and wild-type littermates were injected with LPS (25 mg/kg i.p.). After 4 hours (a) and each time point (b), mice were sacrificed and serum was collected and analyzed for the level of cytokines and chemokines using a Multiplex system (Bio-Rad). Data were analyzed by nonparametric Mann-Whitney test (a) and ANOVA (b) to compare wild type to *NAG*-1^*Tg*/*Lox*^ mice and are representative of two individual experiments.

LPS is a commonly used and well-studied systemic inflammatory agent that signals through the Toll-like Receptor 4 (TLR4) leading to NF*κ*B pathway activation and the subsequent expression of inflammatory cytokines. Treatment of mice with LPS increases the circulating levels of inflammatory cytokines, offering a convenient and suitable assay to determine how alterations in the expression of specific genes modulate the inflammatory response. The concentrations of several inflammatory cytokines were measured in serum from WT and *NAG*-1^*Tg*/*Lox*^ mice 4 hours after treatment with LPS (Figure 1(a)). Serum concentrations of the cytokines IL-6, KC, and MCP-1 were significantly lower in the *NAG*-1^*Tg*/*Lox*^ mice, as compared to WT littermates following LPS treatment. To evaluate the kinetics of cytokine production in the *NAG*-1^*Tg*/*Lox*^ mice, serum was collected at 2, 4, and 6 hours after LPS treatment. At all time points examined, we observed lower levels of IL-6, KC, and MCP-1 in the *NAG*-1^*Tg*/*Lox*^ mice as compared to their WT littermates, with the most dramatic reduction observed for TNF-*α* at 2 hours (Figure 1(b)). No significant differences were observed between the responses of 1377 and 1398 lines to LPS, and only the results from 1398 are reported. No statistically significant difference between male and female (data not shown) was found. These results suggest that *NAG*-1^*Tg*/*Lox*^ mice have a reduced response to inflammatory stimuli and that NAG-1 may have anti-inflammatory activity. 

### 3.2. Macrophages, NAG-1, and NF*κ*B Pathway

**Figure 2:**
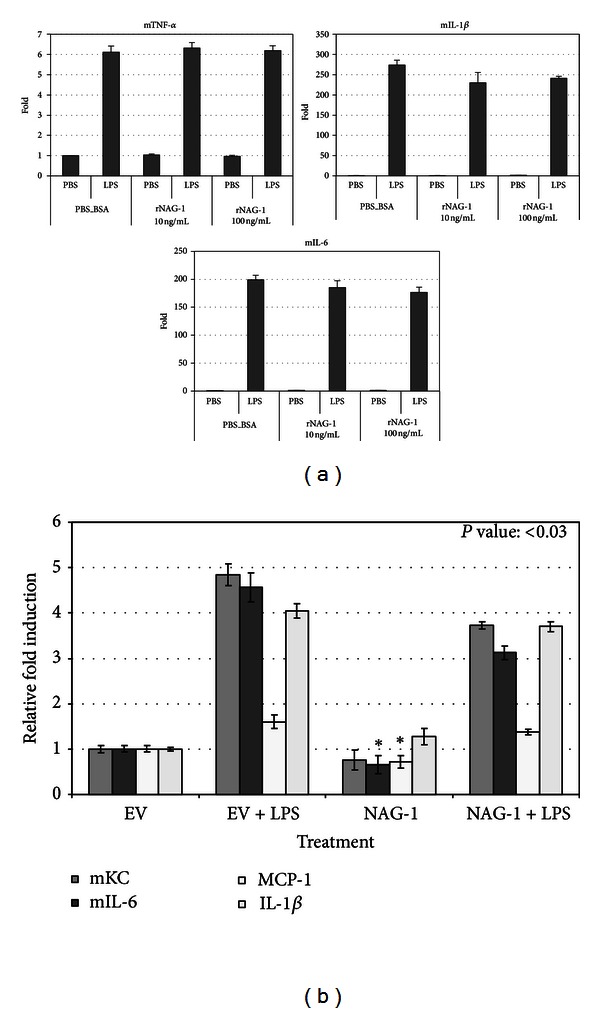
NAG-1 does not inhibit LPS-induced inflammatory cytokine formation in macrophages. (a) RAW 264.7 cells were plated in 60 mm dishes. After 24 hr, cells were treated with NAG-1 protein (R&D) at the concentrations indicated for 5 hrs and then treated with LPS (5 ng/mL) for 20 hrs. RNA was isolated from the collected cells and used for real-time PCR analysis, using primers specific to the genes indicated. The data are expressed as fold increases compared to untreated cells. Error bars represent standard deviation. (b) RAW 264.7 cells were transfected with empty vector (−) or NAG-1 expression vector (+), and then the cells were treated with 1 *μ*g/mL LPS for 6 hours. RNA was extracted and used for real-time PCR analysis, using primers specific to the genes indicated, using m*β*-actin as a control gene. The data are expressed as fold increases compared to untreated cells. Error bars represent standard error.

Activation of the NF*κ*B pathway and the subsequent increase in the production of inflammatory cytokines are important components of the systemic response to LPS. The NF*κ*B pathway also plays an important role in intestinal inflammation and tumor formation [3]. Thus, we examined whether NAG-1 could directly inhibit this pathway. Macrophages were chosen for these experiments, as they are one of the major sources of inflammatory cytokines in response to LPS and are a suitable model system to study the NF*κ*B pathway [8] and previous studies have demonstrated that NAG-1/GDF15 inhibits cytokine production from macrophages [11]. RAW 264.7 cells, a mouse macrophage cell line responsive to LPS, were treated with 10 to 100 ng/mL recombinant mature human NAG-1 protein (rhNAG-1) for 2 hours prior to LPS treatment (5 ng/mL) and incubated for the indicated times. Two different preparations of purified recombinant human NAG-1 protein were used. One preparation, which is reported to have activity in a number of biological systems, contains an N-terminal 6-His tag and the second is tagless [18, 19]. RNA isolated from the cells was used for real-time PCR analysis for several inflammatory mouse cytokines (TNF-*α*, IL-1*β*, and IL-6). As shown in Figure 2(a), while LPS stimulated inflammatory cytokine gene expression in RAW 264.7 cells, pretreatment with rhNAG-1 did not reproducibly inhibit TNF-*α*, IL-1*β*, and IL-6 mRNA levels. We also measured mouse GDF15 mRNA levels using real-time PCR to rule out the possibility that the endogenous protein was contributing towards our phenotype. While mGDF15 mRNA levels increased in response to rhNAG-1, treatment of RAW cells with LPS alone led to an equivalent fold induction of mGDF15. Next, a human NAG-1 expression vector was transfected into RAW 264.7 cells, and cytokine expression was measured using real-time PCR, after 6 hours of treatment with LPS or vehicle. Using primers specific to the human NAG-1 mRNA, we confirmed that human NAG-1 is highly expressed only in the NAG-1 transfected cells. However, as shown in Figure 2(b), there was no difference in cytokine production between empty vector transfected and NAG-1 transfected cells after LPS treatment, despite lower MCP-1 and IL-6 levels in vehicle-treated NAG-1 transfected cells. We also measured mGDF15 using primers specific to the mouse GDF15 mRNA. LPS treatment led to similar induction of mGDF15 in both empty vector and NAG-1 transfected cells (data not shown). Together, these data suggest that NAG-1 does not directly inhibit the TLR4/NF*κ*B pathway.

**Figure 3:**
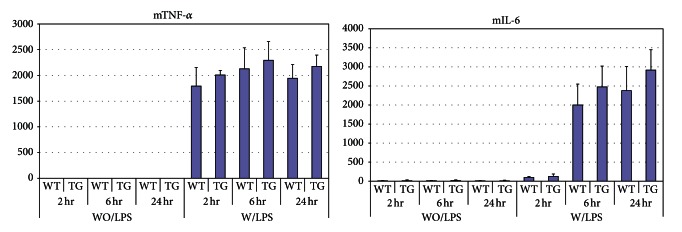
Peritoneal macrophages from WT and *NAG*-1^*Tg*/*Lox*^ mice show no difference in the production of inflammatory cytokines in response to LPS. Peritoneal macrophages were isolated from WT and *NAG*-1^*Tg*/*Lox*^ mice after 3 days of thioglycollate injection and plated on 60 mm dishes. After 24 hrs, cells were treated with LPS (1 ng/mL) and culture media were collected at the indicated time points. The levels of TNF-*α* and IL-6 from 3 dishes for each treatment were measured, in duplicate, using cytokine-specific ELISAs (R&D). The mean ± standard deviation is reported.

To test the hypothesis that expression of the transgene in the developing *NAG*-1^*Tg*/*Lox*^ mouse could alter inflammatory signaling components and explain the reduced response to LPS, peritoneal macrophages were isolated from WT and *NAG*-1^*Tg*/*Lox*^ mice, cultured and treated with 5 ng/mL LPS. The levels of secreted TNF-*α* and IL-6 after LPS treatment were measured in the culture supernatants at the times indicated using cytokine-specific ELISAs. As shown in Figure 3, the production of TNF-*α* and IL-6 in response to LPS was similar in WT and *NAG*-1^*Tg*/*Lox*^ macrophages, indicating that *NAG*-1^*Tg*/*Lox*^ macrophages *ex vivo* do not have impaired TLR4/ NF*κ*B signaling. Furthermore, preincubation of peritoneal macrophages isolated from WT mice with rhNAG-1 did not alter their response to LPS (data not shown). Thus, NAG-1 does not directly inhibit or alter the LPS-activated TLR4/ NF*κ*B pathway in macrophages. The reduction in the inflammatory response to LPS observed in the *NAG*-1^*Tg*/*Lox*^ mouse does not appear to be a consequence of direct inhibition of macrophage-derived cytokine production. 

### 3.3. NAG-1 and Kupffer Cells

**Figure 4:**
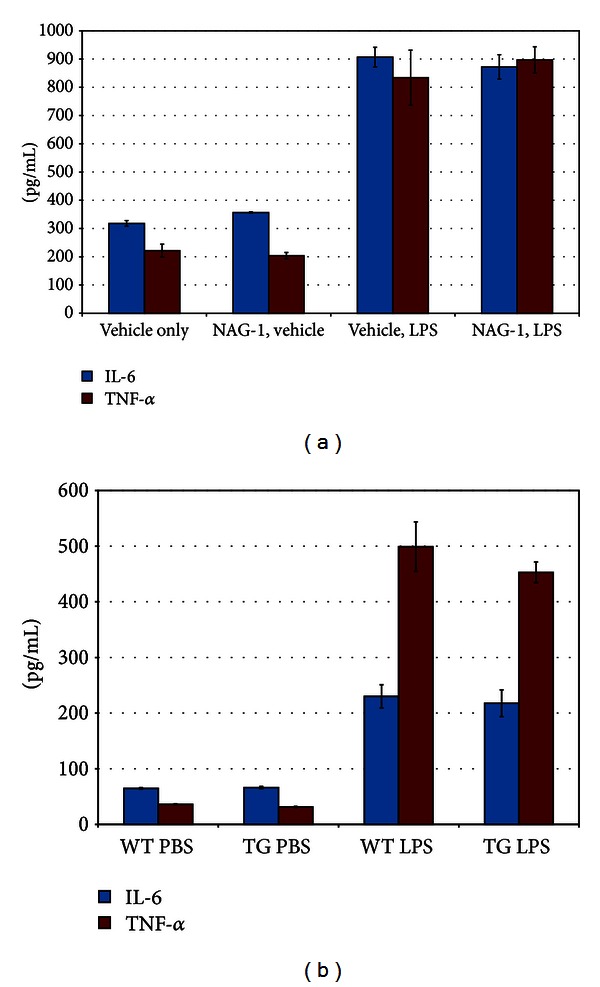
NAG-1 does not affect inflammatory cytokine production from Kupffer cells. (a) Kuppfer cells isolated from the livers of WT mice were plated in triplicate onto 6-well plates and cultured for 24 hrs in RPMI. Media were collected at the beginning of an experiment and cells were then refed and incubated with media containing recombinant human GDF-15 (50 ng/mL) or vehicle for 2 hours and then LPS (10 ng/mL) or vehicle for 6 hours, prior to media collection. TNF-*α* and IL-6 levels in the media were measured in duplicate using cytokine-specific ELISAs. The mean concentrations for each treatment are plotted. Error bars represent standard error. (b) Kupffer cells isolated from the livers of WT or *NAG*-1^*Tg*/*Lox*^ mice were plated in triplicate onto 6-well plates and cultured for 24 hrs in RPMI. Media were collected at the beginning of an experiment and cells were re-fed and incubated with media containing LPS (10 ng/mL) or vehicle and incubated for 6 hours prior to collection of media. TNF-*α* and IL-6 levels in the media were measured in duplicate using cytokine-specific ELISAs. The mean concentrations for each treatment are plotted. Error bars represent the standard error of the mean.

Because Kupffer cells, the resident liver macrophages, are an important source of LPS-induced inflammatory cytokines, we chose to examine their response to LPS *ex vivo*, taking two different approaches. First, we isolated Kupffer cells from WT mice and cultured them in the presence of rhNAG-1 or vehicle prior to LPS treatment. While LPS treatment led to an increase in the media concentrations of IL-6 and TNF-*α* as measured by ELISA, pretreatment with rhNAG-1 had no effect on cytokine production (Figure 4(a)). Taking a second approach, we isolated and cultured Kupffer cells from *NAG*-1^*Tg*/*Lox*^ and WT mice, treated them with LPS, and measured IL-6 and TNF-*α* production. Again, the Kupffer cells responded to the inflammatory stimulus, but there was no difference in the responses between the *NAG*-1^*Tg*/*Lox*^ and WT control cells (Figure 4(b)). 

### 3.4. Leptin and LPS-Induced Inflammation

**Figure 5:**
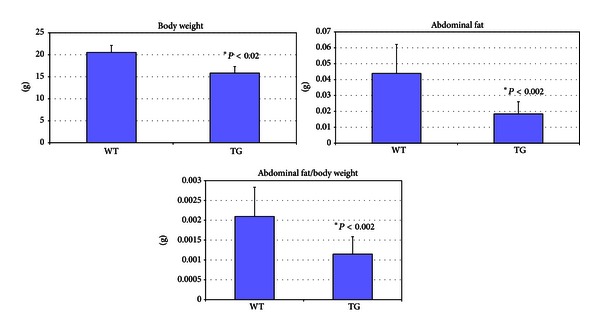
*NA* *G*-1^*Tg*/*Lox*^ mice show lower body weight and less abdominal fat tissue than wild-type mice. 10 female WT and 10 female *NAG*-1^*Tg*/*Lox*^ mice were injected with LPS intraperitoneally. After 4 hrs of LPS treatment, mice were sacrificed and weighed. Blood was collected for serum isolation and abdominal fat tissue was collected, weighed, and snap frozen.

**Figure 6:**
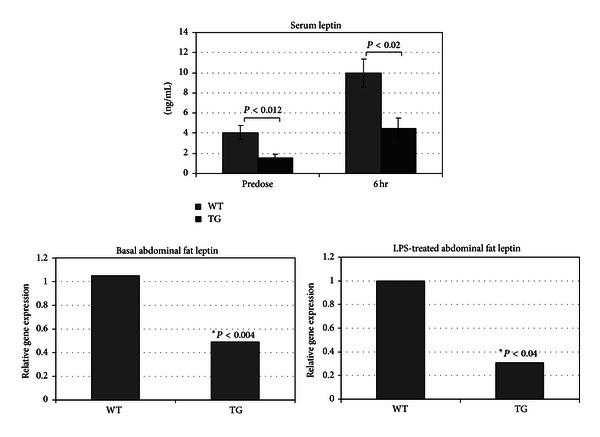
*NA* *G*-1^*Tg*/*Lox*^ mice have lower basal and LPS-induced serum leptin concentrations and abdominal fat leptin expression than WT littermates. The serum collected from the terminal bleeds (Figure 5) was analyzed for leptin using a cytokine-specific ELISA. RNA was isolated from the isolated abdominal fat tissue, which was analyzed for relative expression of mouse leptin mRNA using RT-PCR.

Leptin is a proinflammatory cytokine and appears to be a pivotal mediator of inflammation in mice [20]. *Ob* ^−^/*Ob* ^−^ mice lacking the leptin gene have a reduced inflammatory response to LPS [21]. Leptin expression is increased by LPS treatment and plays a role in LPS-induced increases in inflammatory cytokine formation [22, 23]. Interestingly, *NAG*-1^*Tg*/*Lox*^ mice have reduced fat content as measured by DEXA densitometry (PIXImus) [4]. Based on these findings, we suspected that *NAG*-1^*Tg*/*Lox*^ mice would have less white adipose tissue and lower basal leptin levels. To test this hypothesis, the body weights, abdominal fat, and serum leptin concentrations of WT and *NAG*-1^*Tg*/*Lox*^ mice were measured. The body weights of the *NAG*-1^*Tg*/*Lox*^ were significantly lower than their WT littermates with significantly less abdominal white fat (Figure 5). Likewise, basal serum leptin levels were significantly lower in the *NAG*-1^*Tg*/*Lox*^ mice than their WT littermates (Figure 6). Treatment with LPS increased serum leptin levels in both WT and *NAG*-1^*Tg*/*Lox*^ mice; however, like basal levels, the final leptin level was significantly higher in WT mice than in the *NAG*-1^*Tg*/*Lox*^ mice. The basal expression of leptin as measured by RT-PCR in the abdominal white fat was approximately 2-fold higher in the WT mice than in the *NAG*-1^*Tg*/*Lox*^ mice (Figure 6). We also measured the expression of leptin and various inflammatory cytokines in the abdominal fat collected 4 hours after LPS treatment (Figure 6). While there were no significant differences in any of the inflammatory cytokines measured, relative WT leptin levels were approximately 3-fold higher than in *NAG*-1^*Tg*/*Lox*^ mice. Thus the *NAG*-1^*Tg*/*Lox*^ mice, having less abdominal fat, express lower levels of leptin and have lower serum concentrations of leptin than WT mice, both before and after LPS treatment. 

## 4. Discussion 

In this study, we demonstrate that the transgenic mice expressing hNAG-1 ubiquitously, *NAG*-1^*Tg*/*Lox*^ mice, have less white adipose tissue and a lower serum level of the inflammatory cytokine leptin. These *NAG*-1^*Tg*/*Lox*^ mice have a reduced response to the systemic inflammatory agent LPS. Although NAG-1 is reported to directly inhibit inflammatory cytokine production in macrophages following LPS treatment [20], we could not confirm this inhibition. Kupffer cells and peritoneal macrophages isolated from WT and *NAG*-1^*Tg*/*Lox*^ mice responded similarly to LPS treatment, despite pretreatment with purified recombinant NAG-1 protein from two different commercial sources. In addition, overexpression of NAG-1 in RAW cells did not alter the cytokine production in the presence of LPS. Thus NAG-1 does not directly inhibit the TLR4/NFkB pathway activated by LPS and may not be a direct inhibitor of macrophage cytokine formation, as reported by others [20]. 

If NAG-1 does not inhibit cytokine formation, why do *NAG*-1^*Tg*/*Lox*^ mice have a lower inflammatory response and why are these mice resistant to inflammatory driven intestinal tumors? When DEXA densitometry indicated the only apparent phenotype of *NAG*-1^*Tg*/*Lox*^ mice to be less white adipose tissue, we realized that this difference could be indirectly responsible for the LPS phenotype. We propose NAG-1 to have an indirect effect on suppressing the LPS response, based on the finding that *NAG*-1^*Tg*/*Lox*^ mice have significantly lower levels of white adipose tissue, lower leptin expression in their white adipose tissue, and lower serum leptin levels. While we have no direct experimental evidence that leptin is altering the LPS response in the *NAG*-1^*Tg*/*Lox*^ mice and cannot exclude other factors contributing towards the phenotype, we point to the significant body of the literature showing the importance of leptin signaling in the LPS response. For example, pretreatment of peritoneal macrophages with recombinant leptin leads to a significant increase in LPS-induced cytokine production [15]. Furthermore, leptin deficient *ob* ^−^/*ob* ^−^ mice and fa/fa mice which have reduced leptin receptor expression show a significantly decreased response to LPS [21], and *ob* ^−^/*ob* ^−^ mice are more sensitive to LPS-induced lethality [24]. We were able to detect high levels of leptin receptor in both RAW cells and peritoneal macrophages isolated from WT and *NAG*-1^*Tg*/*Lox*^ mice using RT-PCR (data not shown). Leptin expression is reported to be proportional to total adipose mass, yet the levels per gram of white adipose tissue in our *NAG*-1^*Tg*/*Lox*^ mice are lower than those in WT mice, suggesting an additional level of regulation by NAG-1. NAG-1's ability to lower white adipose and circulating leptin levels is not unique to our transgenic mouse model, as it has also been observed in an xenograft model using DU145 cells expressing NAG-1 [25]. 

Leptin is the critical regulator of intestinal inflammation [26] and a number of studies demonstrate that increases or decreases in the serum levels of leptin can, respectively, increase or decrease the AOM-induced and *APC* ^min⁡^ models of intestinal tumorigenesis [27, 28]. Based on the literature, we propose that the lower white adipose tissue and lower circulating leptin levels may be responsible, in part, for the resistance to intestinal polyps formation observed in the two mouse models of intestinal cancer we previously tested. 

Interestingly, the deletion of NAG-1/GDF15 in *APC* ^min⁡^ mice negates the chemoprevention afforded by sulindac treatment [29], suggesting the human and mouse proteins share some common function. However, untreated GDF15−/− mice on the *APC* ^min⁡^ background do not show an increase in polyp number. GDF15 knockout mice and their WT littermates have similar body, adipose tissue weights, and serum leptin levels (data not shown). Likewise, we found that the GDF15 knockout and WT mice have similar responses to LPS as measured by cytokine levels in the serum (data not shown). Two key points can help explain why the GDF15 knockout mouse does not show the opposite phenotype of the *NAG*-1^*Tg*/*Lox*^ mouse. First, mouse NAG-1/GDF15 is expressed mainly in the liver whereas the *NAG*-1^*Tg*/*Lox*^ mice express human GDF15 constitutively and ubiquitously. Second, the mouse and human proteins share only 57% amino acid identity, making it possible that the proteins have unique functions. Future discovery of the structures and receptors of both mouse and human GDF15 will allow for better understanding of their differences. The findings with the GDF15 knockout mice are consistent with the hypothesis that the difference in the amount of adipose tissue and circulating leptin between the *NAG*-1^*Tg*/*Lox*^ and WT mice is an important determinant of the inflammatory response to LPS. 

## 5. Conclusions

In conclusion, we propose that the lower adipose and hence the lower leptin expression may attenuate the response to LPS treatment and may also be responsible for reduced intestinal tumor formation in *NAG*-1^*Tg*/*Lox*^ mice. This hypothesis is in agreement with the critical role of the leptin in mediating intestinal inflammation and tumor growth. The lower expression of leptin as a result of a reduction in white adipose tissue and the reduced leptin expression within the adipose tissue in the *NAG*-1^*Tg*/*Lox*^ mouse provide one mechanism by which NAG-1 may act to alter inflammation and tumor growth. These results also highlight the link between white adipose tissue mass and inflammation. 

## Abbreviations

GDF15: Growth differentiation factor-15
NAG-1: NSAID-activated gene
*NA**G*-1^*Tg*/*Lox*^ mice: C57Bl6 mice expressing human NAG-1
LPS: Llipopolysaccharide
KC: Keratinocyte-derived cytokine
IL-6: Interleukin-6
MCP-1: Monocyte chemotactic protein-1
TNF-*α*: Tumor necrosis factor-alpha
ELISA: Enzyme-linked immunosorbent assay
*ob*^−^/*ob*^−^: Mice, leptin deleted obese mice
WT: Wild-type, db/db, leptin receptor deleted mice.
